# SEMANTIC RETRIEVAL PATTERNS IN COGNITIVELY HEALTHY OLDER ADULTS

**DOI:** 10.1093/geroni/igad104.2629

**Published:** 2023-12-21

**Authors:** Vaishali Phatak, Elizabeth Vlock, Yunju Im, Jennifer Merickel, Erica Aflagah, Daniel Murman, Jun Ha Chang, Matthew Rizzo

**Affiliations:** University of Nebraska Medical Center, Omaha, Nebraska, United States; University of Nebraska Medical Center, Omaha, Nebraska, United States; University of Nebraska Medical Center, Omaha, Nebraska, United States; University of Nebraska Medical Center, Omaha, Nebraska, United States; University of Nebraska Medical Center, Omaha, Nebraska, United States; University of Nebraska Medical Center, Omaha, Nebraska, United States; University of Nebraska Medical Center, Omaha, Nebraska, United States; University of Nebraska Medical Center, Omaha, Nebraska, United States

## Abstract

Word finding difficulties can be a normative change in aging. The purpose of this descriptive study is to establish metrics of word retrieval patterns in older adults using a semantic fluency task. Forty-five healthy aging, primary English speakers (mean age 75.0; 69% female, mean MoCA = 26.5) participated. Participants were asked “I want you to tell me all the animals you can think of in one minute.” The responses were scored using two novel normalized coefficients. (1) Archetypal Coefficient (AC): frequency proportional to the 60-second frequency. (2) Seeding Coefficient (SC): frequency proportional to both the 60-second frequency and the first-word frequency. Cluster coefficients (CC) also measured adjacency of responses. Participants generated mean of 19.9 words with a low correlation to MoCA (r=0.32). A total of 204 unique animal names including developmental stages (e.g., puppy), sex differences (e.g., rooster), and nicknames (e.g., bunny) were produced. In descending order, the most frequent responses were cat (AC=1.00), dog (AC=0.99), elephant (AC=0.89), horse (AC=0.83), and lion (AC=0.78). SC showed high ranking for archetypal animals (e.g., dog (SC=1.00), cat (SC=0.72)) and animals beginning with the letter “a” (e.g., anteater (SC=0.18), aardvark (SC=0.17)). CC showed greater adjacency for semantic subcategories such as pets (cat-dog) or farm animals (cow-horse), closely related species (mouse-rat) and superordinate labels (bird-fish). Our novel indices and CC revealed semantic retrieval in healthy aging occurs in an organized fashion that includes linguistic (i.e., alphabetical, word frequency) and semantic characteristics of animals, which adds to the current literature on semantic networks.

